# Optimal method for reliable lateral spread response monitoring during microvascular decompression surgery for hemifacial spasm

**DOI:** 10.1038/s41598-023-49008-1

**Published:** 2023-12-07

**Authors:** Kyung Rae Cho, Hyun-Seok Lee, Minsoo Kim, Sang-Ku Park, Kwan Park

**Affiliations:** 1 Department of Neurosurgery, Konkuk University Medical Center, 120, Neungdong-ro, Gwangjin-gu, 05029 Seoul, Republic of Korea; 2 Department of Neurosurgery, Gangneung Asan Hospital, University of Ulsan College of Medicine, Gangneung, 25440 Republic of Korea; 3 Department of Neurosurgery, School of Medicine, Sungkyunkwan University, Seoul, Republic of Korea

**Keywords:** Neuro-vascular interactions, Neurophysiology, Neurological disorders

## Abstract

In this study, we propose an optimal method for monitoring the key electrophysiological sign, the Lateral Spread Response (LSR), during microvascular decompression (MVD) surgery for hemifacial spasm (HFS). Current monitoring methods and interpretations of LSR remain unclear, leading to potential misinterpretations and undesirable outcomes." We prospectively collected data from patients undergoing MVD for HFS, including basic demographics, clinical characteristics, and surgical outcomes. Stimulation intensity was escalated by 1 mA increments to identify the optimal range for effective LSR. We designated the threshold at which we can observe LSR as THR1 and THR2 for when LSR disappears, with high-intensity stimulation (30 mA) designated as THR30. Subsequently, we compared abnormal muscle responses (AMR) between the optimal range (between THR1 and THR2) and THR30. Additionally, we conducted an analysis to identify and assess factors associated with artifacts and their potential impact on clinical outcomes. As stimulation intensity increases, the onset latency to detect AMR was shortened. The first finding of the study was high intensity stimulation caused artifact that mimic the wave of LSR. Those artifacts were observed even after decompression thus interfere interpretation of disappearance of LSR. Analyzing the factors related to the artifact, we found the AMR detected at onset latency below 9.6 ms would be the lateral spreading artifact (LSA) rather than true LSR. To avoid false positive LSR from LSA, we should stepwise increase stimulation intensity and not to surpass the intensity that cause LSR onset latency below 10 ms.

## Introduction

Previous pioneering studies have implicated vascular compression of the facial nerve root exit zone (REZ) in the pathophysiology of hemifacial spasm (HFS)^1^. Neurophysiological investigations have provided insights into the mechanisms underlying the abnormal muscle response (AMR), which appears as a lateral spreading response (LSR), during intraoperative monitoring at microvascular decompression (MVD)^2^. AMR is a term that broadly encompasses any unusual muscle activity. However, it is commonly used interchangeably with LSR in literatures related to HFS. In our study, we introduce a distinct type of AMR that mimics LSR, and we refer to this phenomenon as the "Lateral Spread Artifact" (LSA). In this article, when we mention AMR, it encompasses any abnormal EMG findings observed during MVD surgery, whether they are LSR or LSA. It's worth noting that this usage of AMR as an umbrella term is not as common in the broader literature where LSR is typically used to describe similar phenomena. Since the discovery of this pathognomonic electrophysiological sign, monitoring for LSR is routinely performed during microvascular decompression (MVD) surgery for HFS. LSR is usually considered to indicate successful decompression and cure of disease; however, many factors of LSR still remain unknown. In many cases, LSR disappears before decompression, few minutes after decompression or is consistently present till the end of the surgery. In many cases, intraoperative findings of LSR are not always in line with their clinical outcomes^3^. Thirumala et al. have reported that residual LSR persisted after successful decompression in 17%–20.3% of their patients^2,4^. Another study have revealed that after decompression, 14% and 10% of the patients experienced partial and no relief from LSR, respectively^5^. They insisted that residual LSR is not related to the clinical outcomes; thus, though LSR can be an effective tool for ensuring complete decompression, no predictors of the long-term outcomes have been identified.

Though electrophysiological pathophysiology of HFS is still unclear; however, several hypotheses explaining the pathophysiology exist. Among the most recognized hypotheses are the peripheral demyelination and central nucleus hyperexcitability hypotheses^6–11^. Along with the origin of the disease, the mechanism and interpretation of LSR are also not well understood^12^. The meaning of amplitude and onset latency and the reason for the disappearance of LSR after nerve decompression have not been elucidated. Moreover, there is no specific method for the optimal monitoring or interpretation of LSR. Usually, a 0.3 ms pulse wave of 5–25 mA is used to stimulate the nerve; however, there is no definite consensus on the stimulation parameters and measuring methods^13^. While conventional electromyography (EMG) can typically be recorded as stimulation intensifies until it reaches the supramaximal threshold, the monitoring of LSR can be conducted using a similar approach as other EMG recordings. However, it is worth noting that the electrophysiology of LSR deviates from the typical responses observed in other EMG recordings and exhibits distinctive patterns. During our monitoring of Lateral Spread Response (LSR) in over 5000 cases, we identified certain typical artifact-like patterns that could potentially be conveyed through a route distinct from the standard LSR pathway. This is further elaborated in the discussion and illustrated in Fig. 7. Because surgeons rely on disappearance of LSR as successful decompression and try to seek for other culprit vessels when LSR is not gone after decompression, misinterpretation can lead to unsatisfactory results or unwanted complications. Thus, in this study, we aimed to determine the optimal stimulation method to monitor LSR by prospectively analyzing patients who underwent MVD for HFS and their findings of LSR using different stimulation parameters during the surgery.

## Materials and methods

This prospective study was approved by Konkuk Medical Center institutional review board (KUMC 2021-06-011-003; first registration at 22/06/2021). And registered at Clinical Research Information Service, which is one of World Health Organization approved institution for clinical trials. (KCT0007866; first registration 2/11/2022). All methods were performed in accordance with the relevant guidelines and regulations. We enrolled patients who underwent MVD for HFS at our institution between June 2021 and March 2023. The exclusion criteria were described in supplement material. We obtained written informed consent from each patient.

### Clinical presentation

Data on the following demographic characteristics were collected from the electronic medical records: sex, age at surgery, duration of symptoms, comorbidities (such as hypertension, diabetes mellitus (DM), or other noticeable pathology), and history of botulinum toxin injections for HFS. The patients’ symptoms were evaluated and classified as described in our previous study. Briefly, the Samsung Medical Center grading system (SMC grade) was used to grade the symptoms; accordingly, compression was classified into eight different types^14,15^. SMC grade refers to the severity of spasm in HFS patients. The higher the grade of SMC grade, severity of spasm is severe and interferes quality of life in patients. More details about the SMC grade are described in the supplementary materials. Using magnetic resonance imaging findings, offending vessels were confirmed in the surgical field with a microscope. Indentation and discoloration at the facial nerve REZ were also evaluated. Clinical outcomes immediately, 1 month, and > 3 months after the surgery were recorded. Complications, such as hearing loss, cerebrospinal fluid leakage, and facial palsy were also reviewed.

### Surgical method

A small sub-occipital craniectomy was performed. After going through the dura, the cerebellum was retracted using suction or occasionally a retractor to expose the facial nerve REZ and the vessel compressing the nerve. After a meticulous dissection of the arachnoid membranes, we exposed the facial nerve REZ and the compressing vessel. We then performed the interposition technique by inserting Teflon felt between the REZ and the vessel to create a wider separation between these structures. Decompression was assured when definite offenders are removed from REZ with disappearance of LSR. If there were no LSR or early disappearance of LSR before decompression, 360° examination around the facial nerve REZ was performed to confirm a fully mobilized vascular compression. In case of persistent LSR, we rigorously sought the offending vessel (which could be hidden), although an excessive retraction was not performed to avoid serious complications.

### Evaluation of LSR

After induction of general anesthesia, the patient was positioned laterally with the head fixed on a Mayfield head fixator. Monitoring electrodes were positioned for evaluating the brainstem auditory evoked potential and LSR.

In our experimental setup, subcutaneous bipolar stimulation needle electrodes were utilized, maintaining a consistent 3 cm separation between the anode and cathode. Electrode placement typically situates the anode electrode 2–4 cm distal to the cathode electrode to prevent anodal block^16^. Stimulation was administered with a duration of 0.3 ms. Furthermore, we conducted stimulation on both the upper and lower branches, specifically targeting the marginal mandibular branch and the temporal branch, to comprehensively investigate LSR responses in two distinct directions, LSR detected in lower facial muscles as LSR I and upper facial muscles as LSR II.

We positioned subcutaneous needle electrodes at the following muscle locations: frontalis, orbicularis oculi, orbicularis oris, and mentalis. Notably, the electrodes placed over the frontalis and orbicularis muscles were dedicated to recording responses elicited by upper branch stimulation via the mandibular branch. In contrast, LSR recordings from the oris and mentalis muscles were obtained with stimulation applied at the temporal branch of the facial nerve.

In all cases, an expert neurophysiologist (S.K.P) performed intra-operative neuromonitoring using NIM Eclipse® E4 (Medtronic, MN, U.S.A.).

We evaluated LSR before craniotomy and dura opening. Stimulation intensities were increased from 1 to 30 mA in a stepwise manner until significant LSR was detected in each patient. We gradually increased the stimulation intensity to pinpoint the minimum level at which LSR was detected, which we denoted as THR1. Similarly, we determined the maximum stimulation intensity at which LSR ceased, labeled as THR2. The intensity range between THR1 and THR2 was termed the "optimal range (OR)." To compare the OR of stimulation and supramaximal stimulation intensity, we established a threshold for stimulation intensity surpassing THR2, which was set at 30 mA in our study, and termed it THR30. Similarly, within this framework, we designated the onset latency of the AMR at between range of THR1 and THR2 as LATO (latency in OR), and latency at THR30 as LAT30. We also measured onset latency of AMR that rest after decompression.

LSR was tested during decompression until finish of surgery. Furthermore, we conducted an analysis of the patterns associated with AMR by incrementally increasing the stimulation intensity. Since the amplitude of AMR varies significantly among patients, influenced by factors such as anesthesia, electrode positioning, and individual differences, we opted for an analysis based on the increasing patterns rather than relying on specific, fixed values.

### Statistical analysis

All statistical analyses were performed using the R statistical software (v4.1.1; R Foundation for Statistical Computing, Vienna, Austria). Mean LSR onset latency and amplitude at different stimulation intensities were compared. A paired t-test was used to compare the mean LSR onset latency and amplitude at different stimulation intensities between THR1 and THR2, OR, and THR30. We created a density plot to compare THR1 with THR2 and to compare the onset latency of LAT30 with LAT0. The density plot was generated using R software, with the area under the curves totaling 100%. The density related to the values on the x-axis is represented on the y-axis. Logistic regression of contributing factors that might result artifact and factors influencing clinical outcome was performed. Patient’s demographic factors and surgical factors are analyzed separatively. The *p* value < 0.05 was considered as statistically significant.

## Results

### Basic demographic findings

A total of 153 patients were enrolled during the study period. There were 119 women and 34 men, and their mean age was 55.3 ± 11 years. After excluding one patient with an epidermoid cyst (in accordance with our exclusion criteria), 152 patients were finally evaluated. Left- and right-sided spasms were observed in 74 and 78 patients, respectively. Regarding the severity of spasms, 48 (31.6%), 65 (42.8%), and 39 (25.7%) patients had spasms of grades 2, 3, and 4, respectively. The mean duration between HFS onset and surgery was $59.1\pm 38.8$ months. Hypertension, controlled DM, and a history of botulinum toxin injection for HFS were recorded in 46 (30.3%), 18 (11.8%), and 35 (23.0%) patients, respectively.

The most common offending vessel was the anterior inferior cerebellar artery (n = 72; 48.0%), followed by multiple vessels (n = 49; 32.7%), the posterior inferior cerebellar artery (n = 24; 16.0%), and others (such as the venous offender and vertebral artery [n = 5, 3.3%]). The most common types of compression, under microscopic view, were the perforator (n = 38; 26.8%), and tandem (n = 33; 23.2%) type. Indentation and discoloration of REZ were also observed. The main discoveries are explained in depth and summarized in Table 1.

**Table 1:** Basic demographics of cases in the study.

Age		55.3 ± 11.3
Gender	F	119 (76.8)
	M	33 (21.3)
Side of spasm	Left	74 (48.7)
	Right	78 (51.3)
Grade (%)	2	48 (31.6)
	3	65 (42.8)
	4	39 (25.7)
Duration of symptom		59.1 ± 38.8
Pre-BTX (%)	1	35 (23.0)
HTN (%)	1	46 (30.3)
DM (%)	1	18 (11.8)
Offender (%)	AICA	72 (48.0)
	PICA	24 (16.0)
	others	5 ( 3.3)
	Multiple vessels	49 (32.7)
Type (%)	Arachnoid	20 (14.1)
	Perforator	38 (26.8)
	Loop	15 (10.6)
	Medial	11 ( 7.7)
	Tandem	33 (23.2)
	Sandwich	18 (12.7)
	Encircling	4 ( 2.8)
	Branch	3 ( 2.1)
Indentation (%)	1	89 (58.6)
	2	31 (20.4)
	3	32 (21.1)
Discoloration (%)	1	80 (52.6)

Basic demographics, previous medical history, and surgical findings of cases in the study are described. Only positive findings of previous medical history, and discoloration is described.

*BTX* Botulinum toxin, *HTN* Hypertensio, *DM* Diabetes mellitus, *LSR* Lateral spread response, *AICA* Anterior inferior cerebellar artery, *PICA* Posterior inferior cerebellar artery.

### Clinical outcome

The spasms disappeared immediately in 117 (77.0%) patients; conversely, 35 patients (23.0%) complained of > 10% residual spasms (subjectively). Fourty-nine patients were followed-up at 1 month postoperatively: nine exhibited residual spasms at that time. Furthermore, 120 patients were followed-up at three months postoperatively; among these, 110 (91.7%) exhibited improvements in spasms and 10 (8.3%) exhibited residual or aggravated spasms. Among those who experienced an immediate improvement, six patients exhibited aggravated spasms during follow-up, albeit at a severity that was lesser than that preoperatively: four complained of 30% residual spasms and two complained of > 50% residual spasms. Furthermore, only four immediate non-responders had residual spasms at the last follow-up (< 30% and > 70% residual spasms in three and one patients). The factors potentially related to clinical outcomes were analyzed and are described in Table 2.

**Table 2:** Logistic regression predicting outcome.

	Crude OR (95% CI)	Crude *P* value	Adj. OR (95% CI)	Adj. *P* value	Sig
Previous BTX	4.62 (1.44, 14.85)	**0.01**	11.63 (0.35, 390.04)	0.171	
Gender	0 (0, Inf)	0.993	0 (0, Inf)	0.995	
Age	1.06 (1, 1.13)	0.053	1.13 (0.95, 1.34)	0.16	
Side of spasm	2.28 (0.67, 7.76)	0.186	6.08 (0.31, 120.66)	0.237	
Grade of spasm (ref: grade 2)					
Grade 3	7.55 (0.92, 61.81)	0.059	20.44 (0.49, 847.34)	0.112	
Grade 4	3.92 (0.39, 39.24)	0.246	1.73 (0.03, 101.25)	0.793	
Hypertension	1.03 (0.3, 3.52)	0.967	0.15 (0.01, 2.32)	0.175	
Diabetes	1.4 (0.28, 6.88)	0.681	6.17 (0.13, 292.33)	0.355	
LSR finding (ref: not seen)					
Resolved after decompression	1.33 (0.35, 5.11)	0.675	1.41 (0.07, 27.6)	0.823	
Remained after decompression	0 (0, Inf)	0.993	0 (0, Inf)	0.998	
Offender: ref. = AICA					
PICA	1.22 (0.22, 6.73)	0.821	9.79 (0.31, 309.77)	0.196	
Others	0 (0, Inf)	0.993	0 (0, Inf)	0.999	
Multiple vessels	1.87 (0.54, 6.51)	0.325	5.85 (0.15, 225.64)	0.343	
Type: ref. = arachnoid					
Perforator	2.88 (0.31, 26.51)	0.351	0.42 (0.01, 30.9)	0.69	
Loop	0 (0, Inf)	0.993	0 (0, Inf)	0.997	
Medial	4.22 (0.34, 52.9)	0.264	0.71 (0.01, 84.19)	0.888	
Tandem	1.23 (0.1, 14.46)	0.872	0.32 (0, 72.26)	0.677	
Sandwich	2.38 (0.2, 28.67)	0.496	4.06 (0.03, 581.84)	0.58	
Encircling	0 (0, Inf)	0.996	0 (0, Inf)	0.999	
Branch	0 (0, Inf)	0.997	0 (0, Inf)	0.999	
Indentation: ref. = grade1					
Grade 2	2.05 (0.54, 7.81)	0.293	33.07 (0.69, 1590.92)	0.077	
Grade 3	1.43 (0.34, 6.09)	0.628	13.16 (0.26, 654.93)	0.196	
Discoloration	0.53 (0.17, 1.71)	0.291	0.08 (0, 1.59)	0.098	
Artifact	0.84 (0.26, 2.72)	0.776	0.14 (0.01, 2.39)	0.172	

The factors related to clinical outcome at more than 1 months of follow ups. Only use of botulinum toxin was seem to related in unvariate analysis but no factors were significantly related in multivariate analysis.

*BTX* Botulinum toxin, *AICA* Anterior inferior cerebellar artery, *PICA* Posterior inferior cerebellar artery.

After surgery, 20 patients had facial palsy (severity of grades 2, 3, and 4 in six, eight, and six patients, respectively). Among these, 15 experienced an improvement, but palsy persisted in the remaining five patients at the last follow-up (severity of grades 2 and 3 in four and one patients, respectively). Postoperative transient hearing loss was observed in 12 patients, whereas one patient became completely deaf postoperatively.

### Findings of LSR

LSR disappeared immediately after dura opening in 41 patients (27.0%), while it disappeared after dissection of the neurovascular conflict and insertion of a Teflon felt in 105 patients (69.1%). Six patients (3.9%) experienced delayed disappearance. However, in all patients, LSR disappeared during the MVD surgery when stimulated in between OR.

As the primary findings from the stepwise increase in stimulation intensity, we observed that the onset latency of AMR shortened with increasing intensity. Simultaneously, the amplitude of AMR increased with higher intensity, but the amplitudes observed at higher than THR2 were sometimes smaller or occasionally larger, differing from what can be seen during AMR at OR. To evaluate the characteristics of this wave, we monitored AMR at two different intensities: the stimulation intensity that produced the maximal AMR amplitude during incremental increases of stimulation at OR, and the high stimulation intensity labeled as "THR30." The term AMR is used to encompass every pathologic wave observed during electrophysiologic monitoring, including not only LSR but also waves mimicking LSR, referred to as LSA.

**Figure 1:**
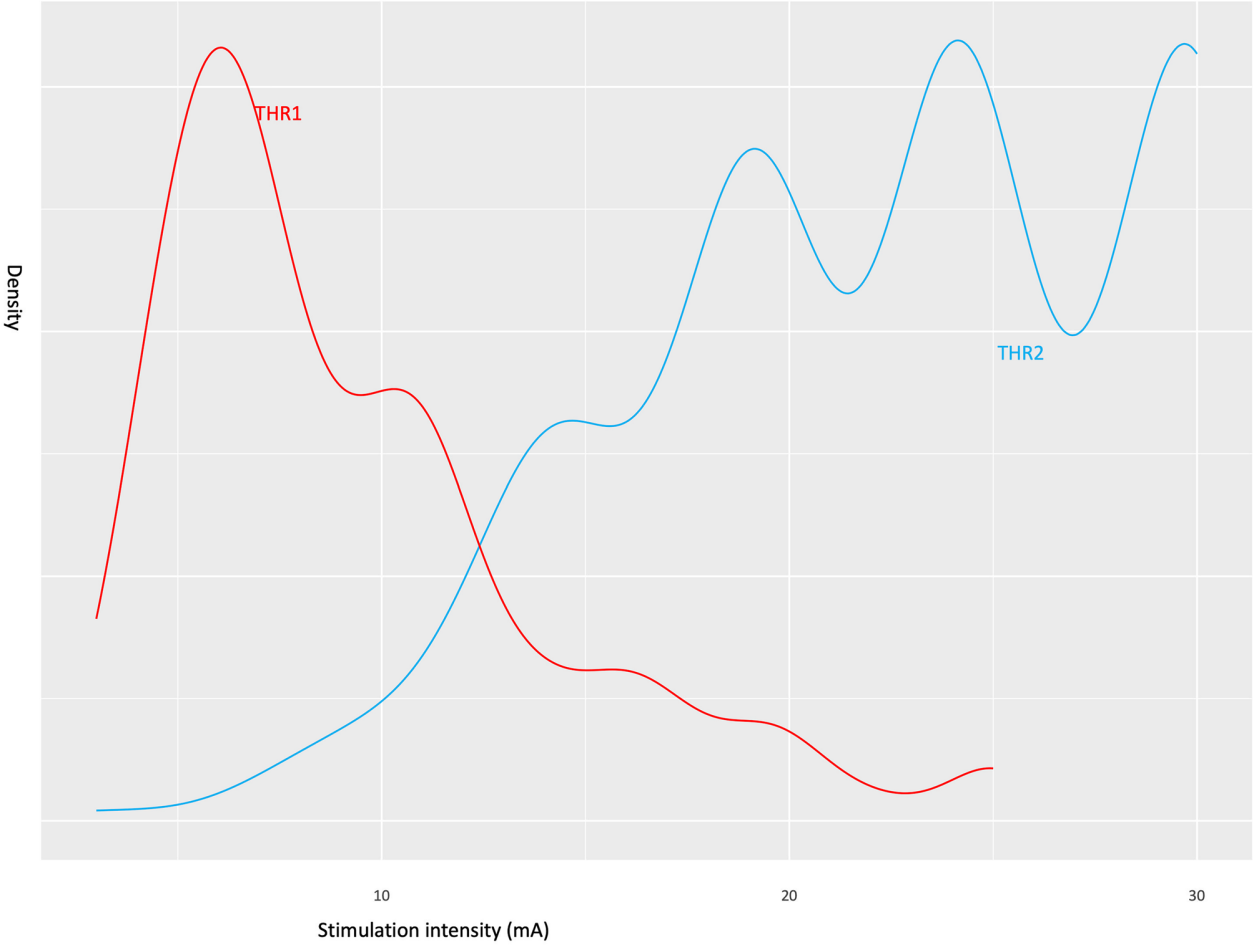
This density plot illustrates the optimal stimulation range in our study. THR1, the threshold where significant abnormal muscle response (AMR) initiation occurred, measured at 8.2 ± 5.5 mA, while THR2, the upper threshold where AMR disappeared or significantly decreased, registered at 22.4 ± 6.1 mA.

**Figure 2:**
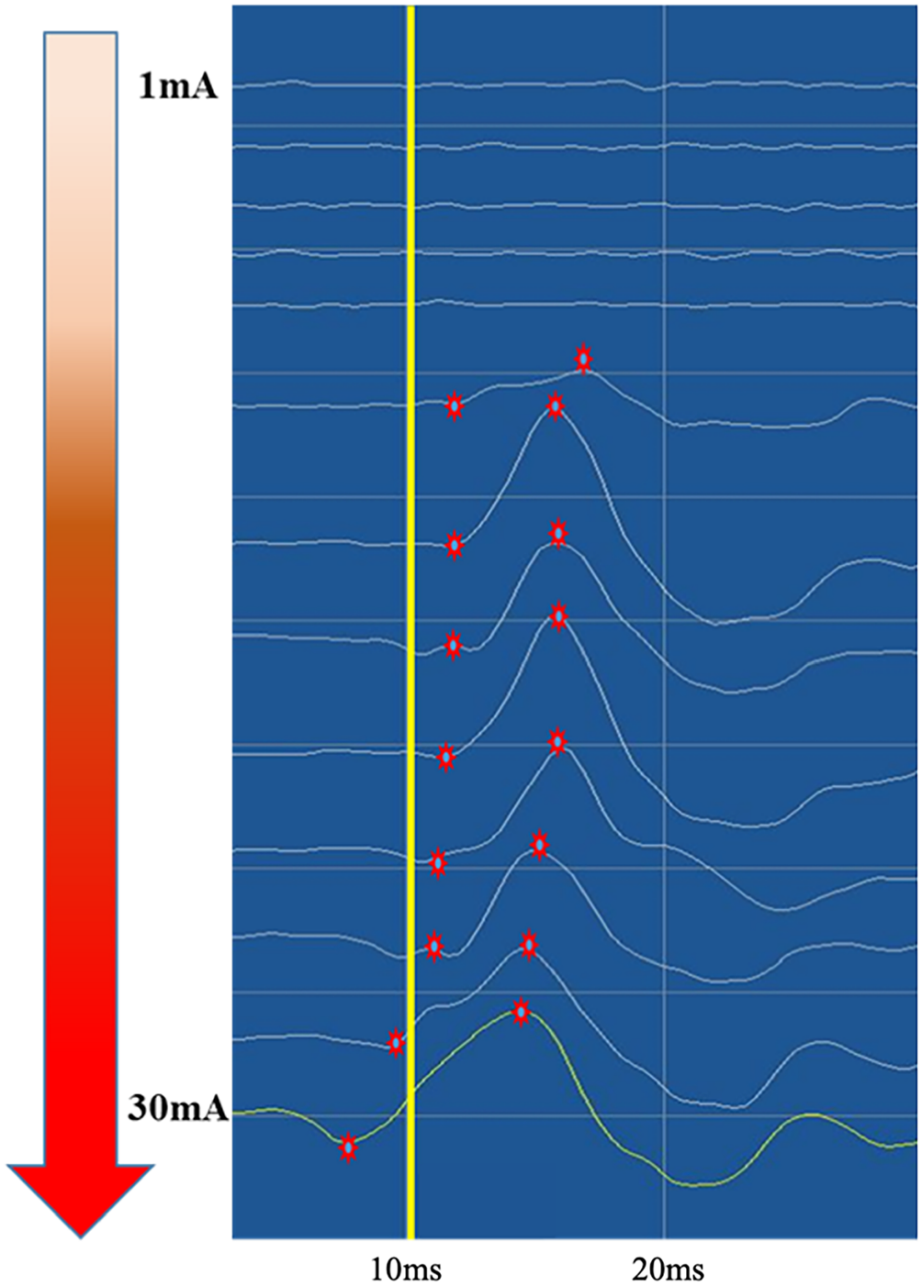
This figure illustrates the relationship between stimulation intensity, abnormal muscle response (AMR), and onset latency. Notably, we delineate the critical point of discrimination between genuine lateral spread response (LSR) and artifact, establishing it at 9.6 ms based on our study findings.

**Figure 3:**
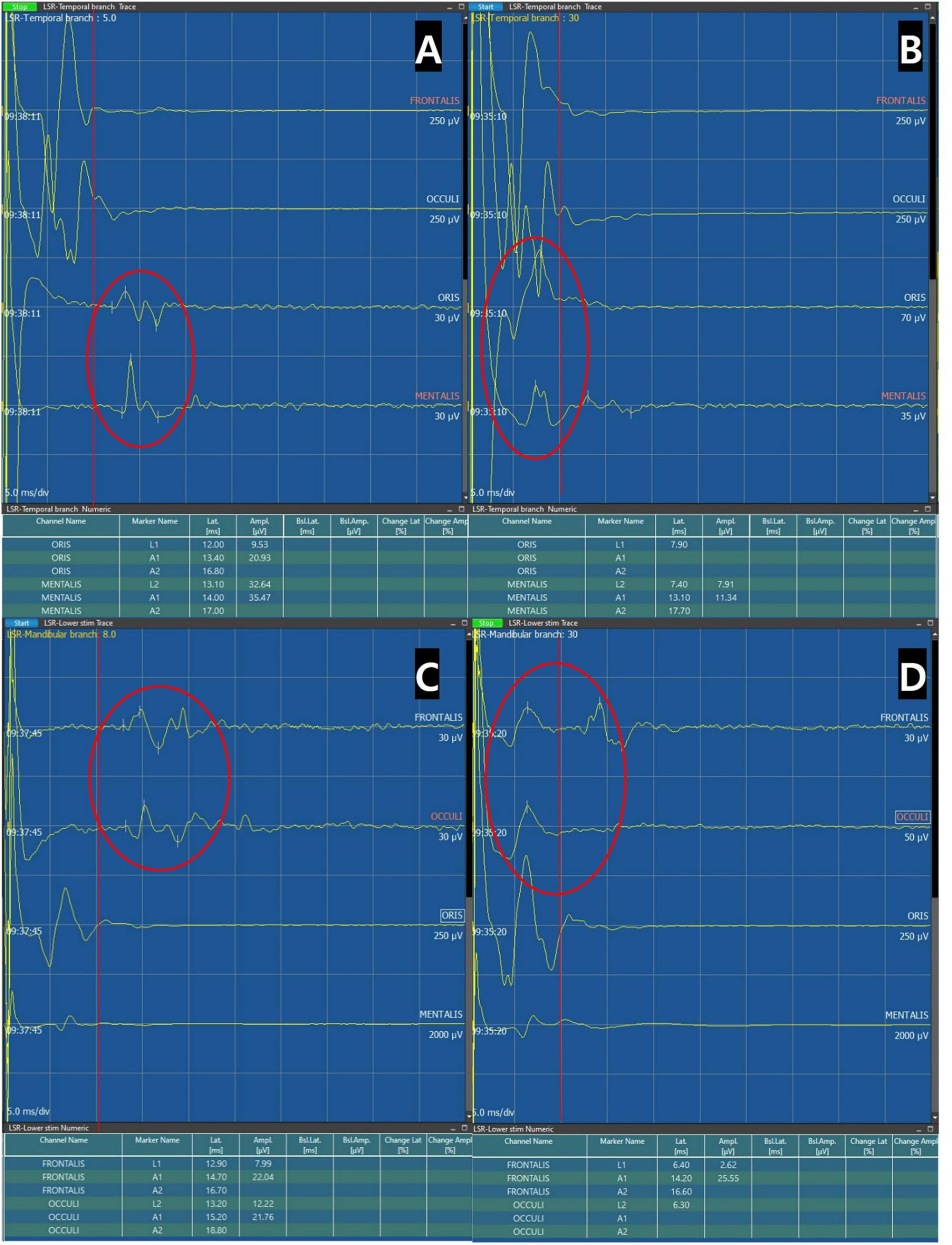
Upper branch stimulation to detect LSR at lower facial muscles (LSR I, **A, B**) and lower branch stimulation to detect LSR at upper facial muscles (LSR II, **C, D**). Lateral spread artifact is found when stimulation of high intensity (30 mA) is applied. (**B, D**) The red circle represents abnormal muscle responses. When the onset latency is shorter than 9.6 ms (as observed in our study), it can be considered a false positive LSR, LSA.

Our primary findings during the 1 mA stepwise escalation of the stimulation intensity were as follows: (1) LSR generally occurred at stimulation intensities between a lower threshold, THR1 of $8.2\pm 5.5$ mA and an upper threshold, THR2 of $22.4\pm 6.1$ mA. (The density plot was described to show range of THR1 and THR2, Fig. 1) (2) The onset latency of LSR, at which the most prominent waveform of a high amplitude is observed was stimulated between OR and mean latency was $11.4\pm 1.6 msec$; the onset latency period shortened as the stimulation intensity increased (Fig. 2). (3) While applying stimulation intensities greater than the upper threshold, THR30, AMR with smaller amplitudes and shorter onset latency was observed in some patients, their mean latency was $7.7\pm 1.9 msec$; these were termed as LSAs (Fig. 3).

**Figure 4:**
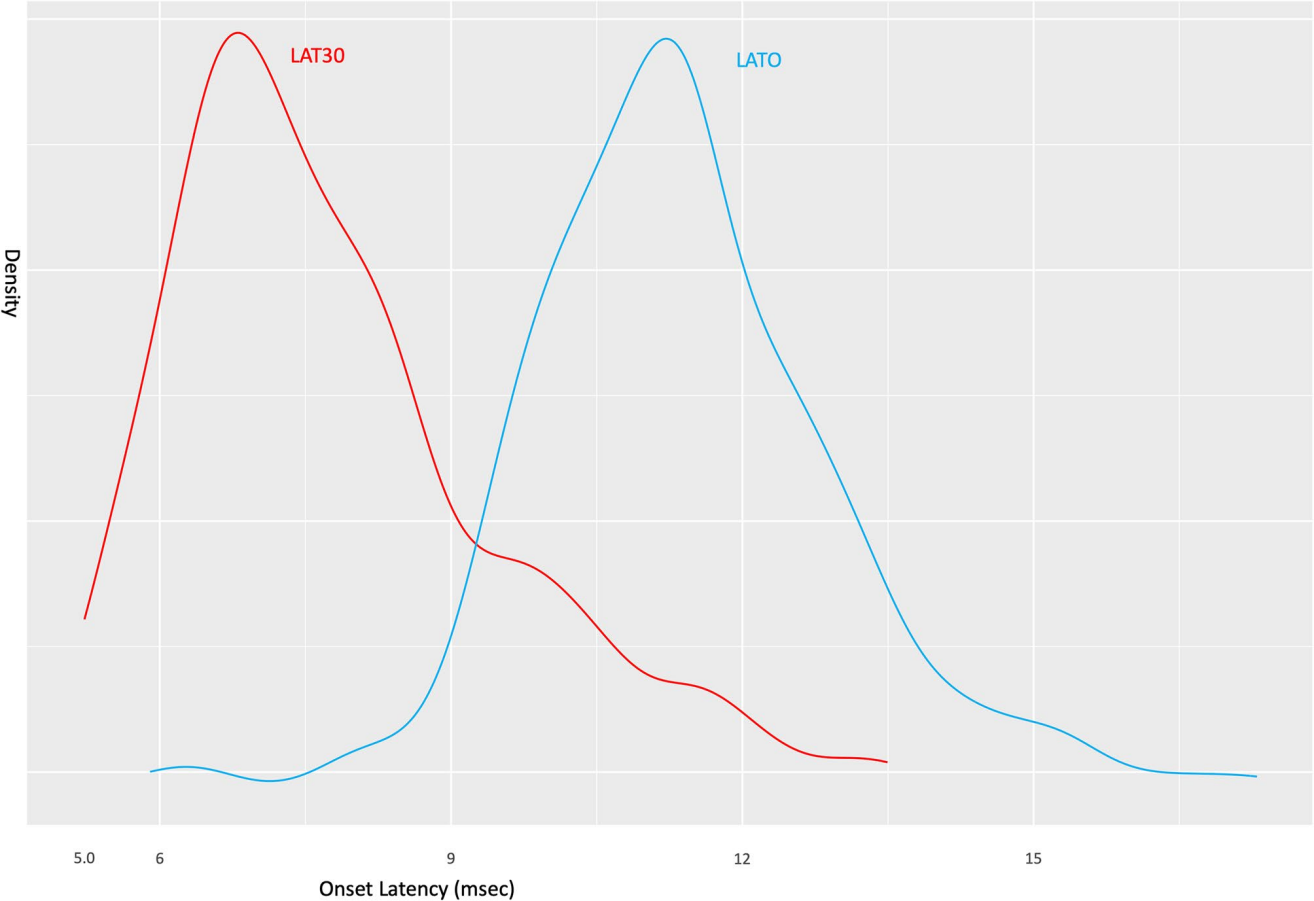
This density plot depicts the onset latency for the optimal range stimulation group (LATO) and the 30 mA stimulation group (LAT30). Notably, LAT30 exhibited a significantly shorter onset latency, and the demarcation between LAT30 and LATO occurred at approximately 9.6 ms, as observed in this study.

Thereafter, we compared the OR and THR30 in terms of the onset latency, amplitude, and pattern of AMR. In our cohort, every patient showed AMR before surgery. Before decompression, definite artifact which can be easily distinguished with LSR was observed in 7 cases with THR30 while no significant artifact was observed in OR. However, after decompression, AMR seen before decompression was completely disappeared in OR in every patient. However, when stimulated with THR30, 99 patients (65.1%) still exhibited the AMR, which differed from LSR seen at OR. The onset latency of AMR was significantly shorter at THR30 than OR (7.75 vs. 11.36 ms; *p* < 0.001) and when the onset latency was detected as shorter than 9.6 ms, it can be assumed to be LSA. (The density plot was drawn to describe difference in onset latency between LATO and LAT30, Fig. 4).

Because these artifacts were not found at OR, they were believed to be “high intensity stimulation” related artifacts, LSA. In conclusion, the presence of shorter latency waves exclusively under high stimulation intensity, absent during low-intensity stimulation, led us to categorize them as LSA. We couldn't identify any factors associated with the presence of LSA, except for the increase in stimulation at THR30. (Table 3).

**Table 3:** Logistic regression predicting outcome.

	Crude OR (95% CI)	Crude *P* value	Adj. OR (95% CI)	Adj. *P* value	Sig
Previous BTX	1.45 (0.64, 3.31)	0.374	1.16 (0.42, 3.17)	0.776	
Gender	0.66 (0.3, 1.46)	0.305	0.63 (0.24, 1.67)	0.356	
Age	1 (0.97, 1.03)	0.821	0.98 (0.94, 1.02)	0.407	
Side of spasm	1.29 (0.66, 2.52)	0.455	1.62 (0.63, 4.12)	0.314	
Grade of spasm (ref: grade 2)					
Grade 3	1.12 (0.52, 2.41)	0.773	1.2 (0.48, 3)	0.703	
Grade 4	1.9 (0.76, 4.78)	0.173	1.63 (0.51, 5.15)	0.407	
Hypertension	1.33 (0.63, 2.79)	0.451	1.12 (0.45, 2.8)	0.808	
Diabetes	1.08 (0.38, 3.06)	0.884	1.71 (0.48, 6.06)	0.407	
LSR finding (ref: not seen)					
Resolved after decompression	0.44 (0.19, 1.01)	0.054	0.54 (0.2, 1.47)	0.227	
Remained after decompression	0.28 (0.05, 1.64)	0.158	0.27 (0.04, 2.09)	0.21	
Offender: ref. = AICA					
PICA	1 (0.39, 2.6)	1	0.93 (0.27, 3.24)	0.909	
Others	0.9 (0.14, 5.73)	0.911	0.51 (0.02, 14.93)	0.694	
Multiple vessels	1.5 (0.69, 3.28)	0.31	1.46 (0.39, 5.52)	0.578	
Type: ref. = arachnoid					
Perforator	0.82 (0.27, 2.47)	0.729	0.88 (0.25, 3.12)	0.84	
Loop	1.83 (0.43, 7.84)	0.413	2.75 (0.44, 17.39)	0.281	
Medial	1.17 (0.26, 5.33)	0.842	1.64 (0.26, 10.22)	0.598	
Tandem	1.78 (0.55, 5.77)	0.338	2.17 (0.38, 12.45)	0.386	
Sandwich	1.05 (0.28, 3.86)	0.944	1.04 (0.18, 6.09)	0.967	
Encircling	2 (0.18, 22.8)	0.577	3.15 (0.23, 42.96)	0.389	
Branch	1.33 (0.1, 17.28)	0.826	0.99 (0.05, 21.6)	0.996	
Indentation: ref. = grade 1					
Grade 2	1.86 (0.75, 4.63)	0.18	1.66 (0.49, 5.63)	0.415	
Grade 3	1.43 (0.6, 3.37)	0.419	1 (0.33, 3.06)	1	
Discoloration	1.4 (0.72, 2.73)	0.324	1.32 (0.51, 3.45)	0.566	

The factors related to artifact seen after decompression. There were no factors that related to occur artifact after decompression. In this table, only patient factor and surgical factor are described.

*BTX* Botulinum toxin, *AICA* Anterior inferior cerebellar artery, *PICA* Posterior inferior cerebellar artery.

**Figure 5:**
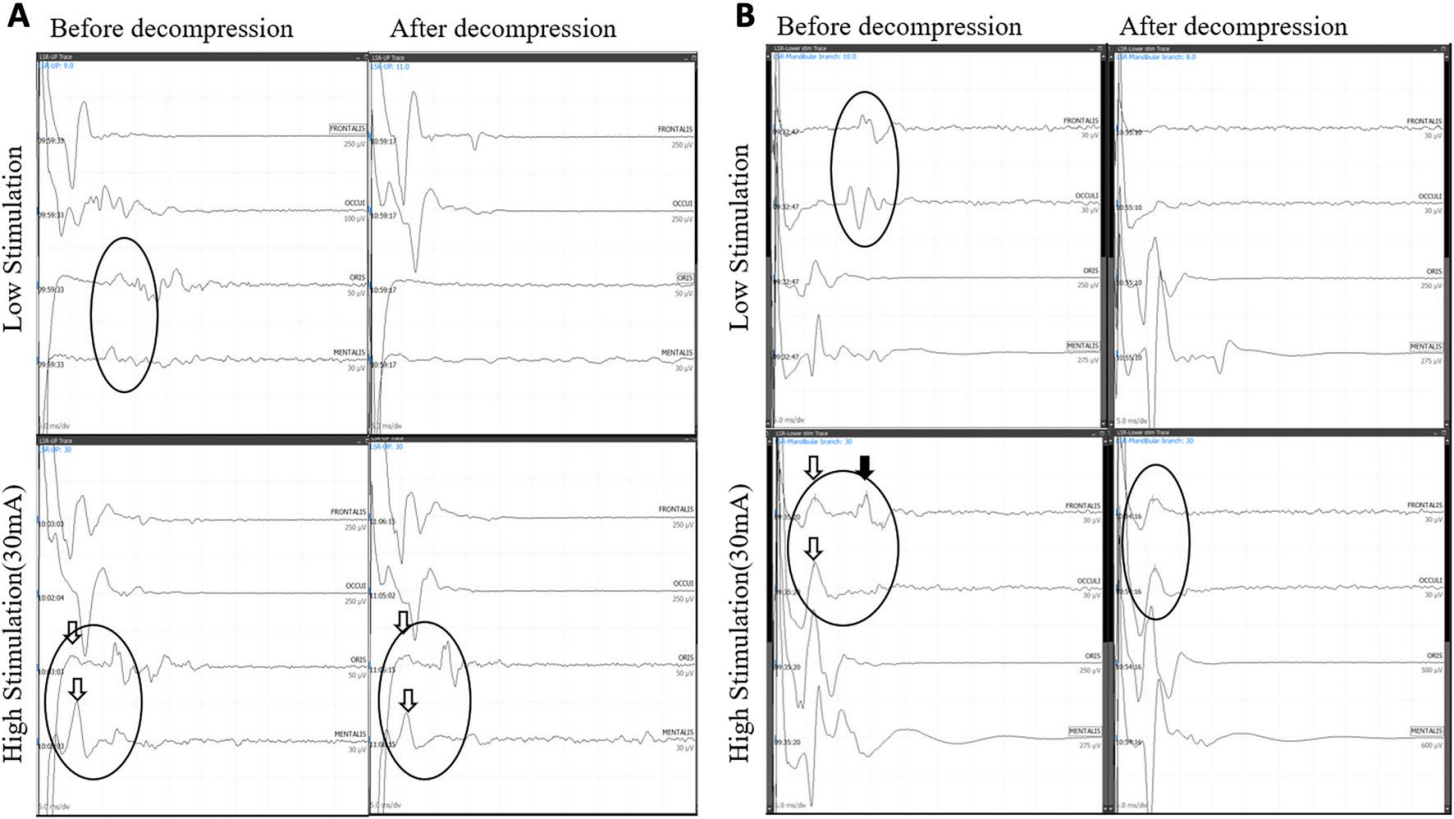
Comparison between the Optimal Range (OR) stimulation and the high amplitude (THR30) stimulation before and after decompression is shown in (**A**) (LSR I) and (**B**) (LSR II). In the OR, LSR disappeared after decompression, while in THR30, residual waves persisted. The black arrow may represent a true LSR or an artifact, whereas the blank arrow indicates a clear artifact that persists after decompression.

Moreover, the LAT30 was even shorter after decompression than that before decompression (7.11 vs. 7.75 ms; *p* < 0.01; Fig. 5).

**Figure 6:**
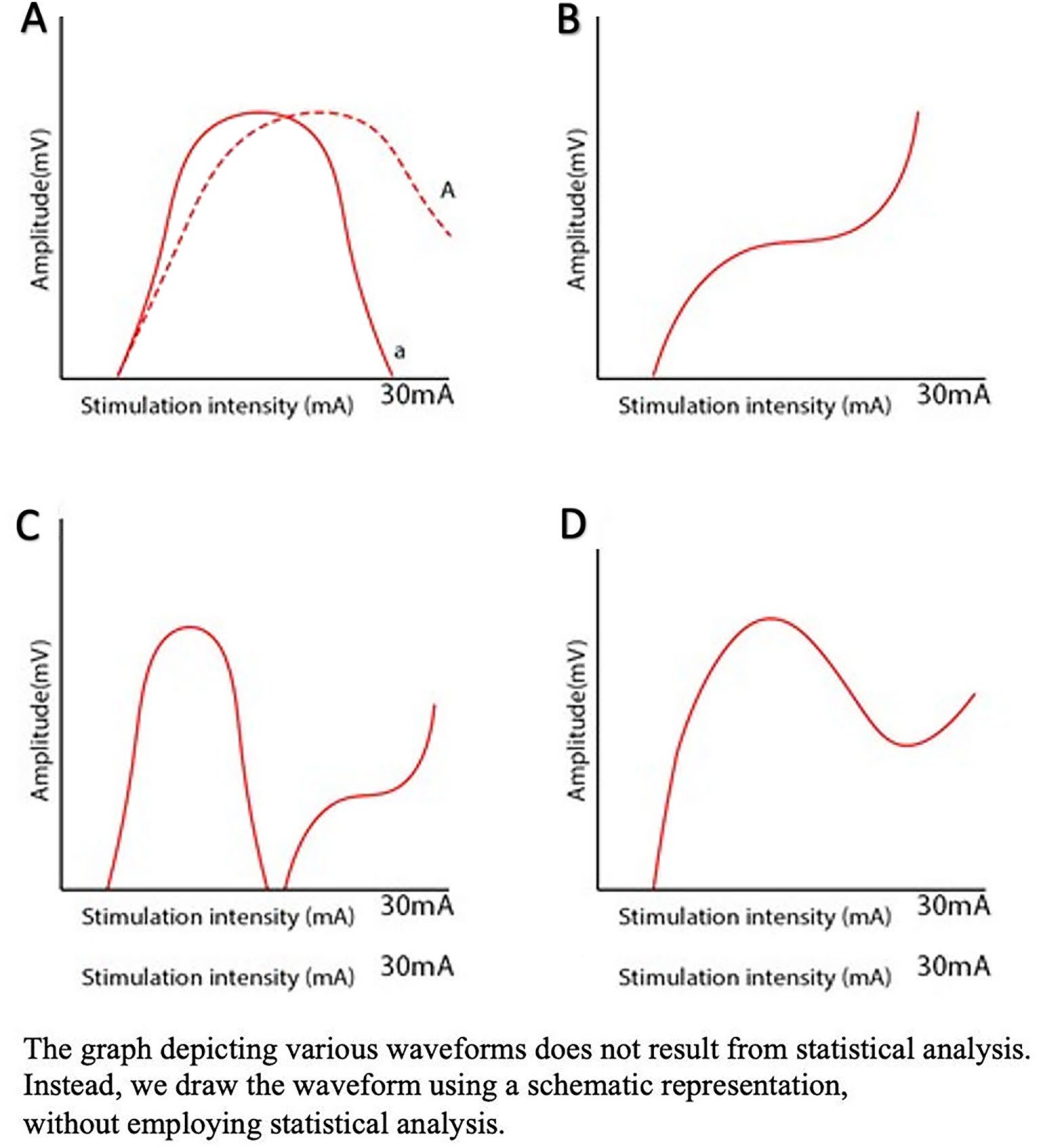
Different patterns of lateral spread response (LSR) waveforms that can be measured during monitoring. Type **A** is the most common, followed by types **B, C**, and **D**. We assume that waves **B, C**, and **D** are summations of the LSR and lateral spread artifact waves.

As stimulation intensity increases, the amplitude of AMR shows four different types of patterns, estimated to be mixed with LSR and LSA. The details of these patterns are described in the supplementary materials and illustrated in Fig. 6.

When we evaluated the factors predicting the LSA at THR30, there were no significant related factor in age, sex, side of spasm, grade, duration of symptoms, and previous medical history. Also, there were no related risk for LSA with surgical findings of offending vessel, compression type, indentation, or discoloration of compression site. When we analyzed if patients with LSA affect clinical outcome, there were no significant relation between the presence of LSA and clinical outcome.

## Discussion

In this study, we found an optimal range of stimulation intensity for measuring LSR. Because OR may vary between each patient, anytime we monitor for LSR we should increase intensity to find THR1 and 2 and stimulate between this “optimal range”. The sensitivity of electrophysiological monitoring was positively related to an increasing stimulation intensity in general. Many examiners increase stimulation intensities for LSR or routinely stimulate with a higher intensity, because there are no studies or guidelines on optimal stimulation for LSR detection. To the best of our knowledge, this is the first study to suggest that stimulation intensities must not exceed the optimal range and that the range can be represented by shortening of onset latency. This onset latency might be different by stimulation methods because we place a cathode at the distal end of the facial nerve for more sensitive detection of LSR, but onset latency might extend because the length of nerve routes is extended^16^.

With incremental increase in stimulation, the onset latency period of LSR was shortened. When stimulation to a nerve is increased, the velocity of nerve conduction and amplitude of nerve action potential may increase, while the latencies may decrease; a similar mechanism can be observed in the facial nerves. Thus LSR may be detected faster regardless of the mechanism of LSR^17^.

Other than shortening of onset latency, we found patterns of AMR by increasing stimulation intensities. As intensity increases high enough, there seems to appear another pattern of AMR. When interpreting EMG, we recorded action potentials delivered to specific muscles as waveforms. We could hypothesize that the AMR waveforms we obtain within the typical threshold represent genuine LSR. However, once the threshold is exceeded, LSA combines with the LSR waveform. According to our study, these waveforms become summated into higher-amplitude waves at higher stimulation intensities or sometimes even smaller in amplitude. Based on these patterns, we can assume that there are artifacts that spread through the skin or routes other than the expected route of LSR in THR30. Compared to LSR, LSAs are detected at higher stimulation intensity and may have smaller amplitudes individually. However, when summated with LSR, they can produce amplitudes larger than genuine LSR. However, when LSR disappears and only LSA remains, smaller amplitudes are observed. Thus, LSR disappearance was observed after successful decompression when stimuli were applied within an effective range. However, when stimuli of > 30 mA were applied, LSAs were observed even after decompression, which masked the real disappearance of LSR.

We acknowledge that the amplitude of AMR can vary considerably among patients, making it challenging to pinpoint specific amplitude values. However, in our study, we focused on evaluating the changing patterns of waveforms for each patient and categorized them into four distinct patterns.' Pattern A was the most frequently observed pattern; this indicates that true LSR disappears at a specific intensity, but LSA does not appear below stimulation of 30 mA or appears with a small amplitude that does not affect the disappearing waveform of LSR. Pattern B was the second most common pattern; this indicates that LSA increases with increasing stimulation intensity and combines with the LSR wave to present an exponential increase. Other pattern types can also be explained using the hypothesis that LSA is observed on application of stimulation intensities higher than the optimal intensity that evokes LSR.

Before decompression, only THR30 resulted in LSA; however, due to overlapping of LSR with the LSA and a summation of their amplitudes, distinguishing LSAs from complex waveforms was not possible (unless the LSAs are distinguishable from LSR, as in pattern C). However, after decompression, LSR disappeared and only LSAs were seen when THR30 was applied, indicating that the LSAs overlapped with the true LSR.

**Figure 7:**
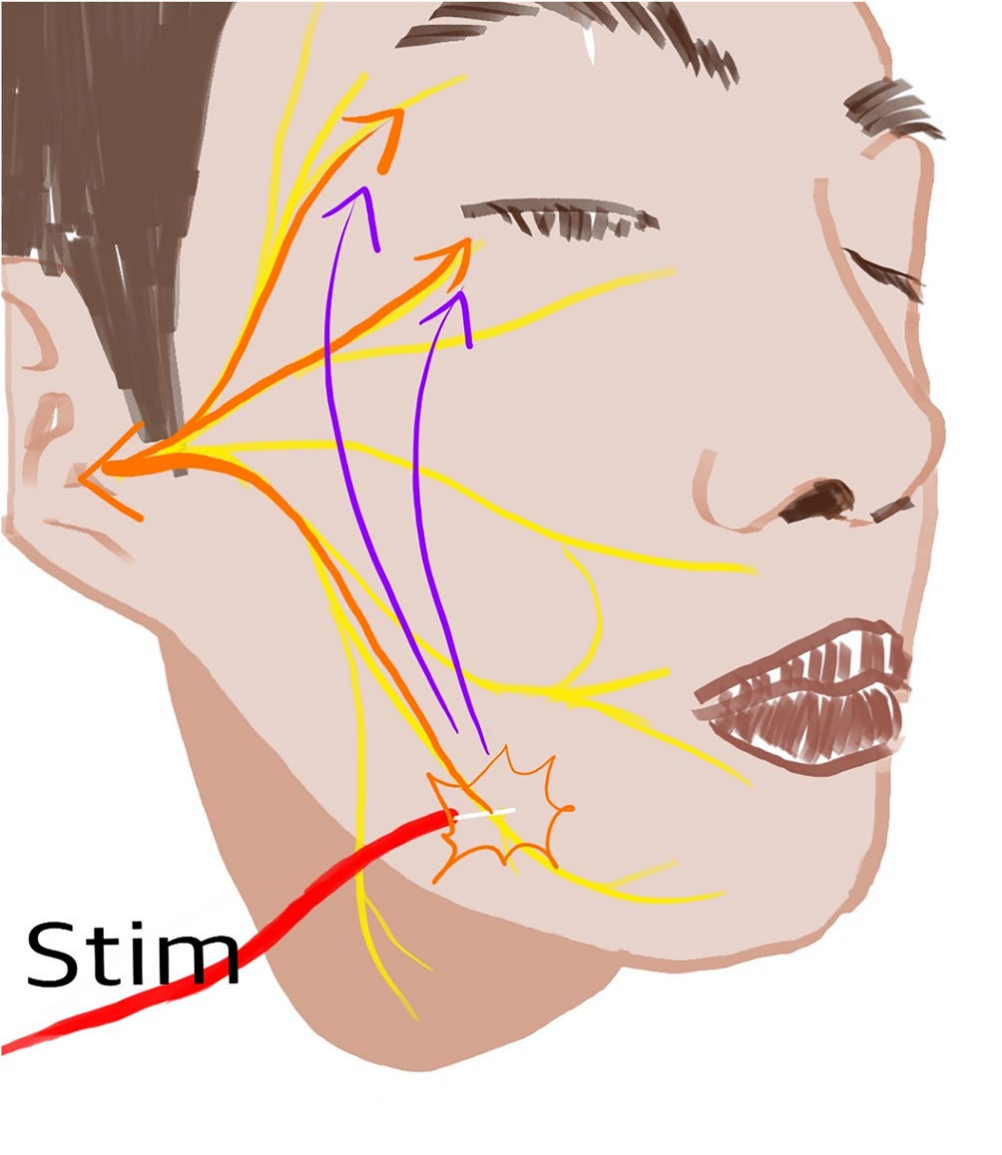
Schematic representations of the lateral spread response (LSR) route (orange arrow) and the lateral spread artifact (LSA) route (purple arrow). LSA directly spread through the skin or other branches of the facial nerve fibers; it makes LSR detection difficult. The figure was drawn by KRC, the author of this literature.

From these findings, we could conclude that there is an optimal range of stimulation that can be used to detect LSR effectively for successful decompression. However, except for LSR in pattern C, we could not distinguish LSR from LSA owing to the continuously changing stimuli-LSR wave patterns and the fact that the stimulation intensity affected the waves of both LSR and LSAs. Hence, we focused on the shortening of LSR onset latency with increasing stimulation intensity. We speculated whether LSR as well as LSA are delivered faster when a high-intensity stimulation is applied and the onset latency is shortened. The mechanisms of LSR and HFS remain unelucidated; however, we hypothesized LSAs found at high stimulation intensities may spread through the skin or directly from the stimulated branch to the affected branch of the facial nerve and not along the pathway of the original LSR (Fig. 7). We also contemplated the reason why LSAs detected at high-intensity stimuli had smaller amplitudes. We hypothesized the physiological mechanisms of LSR and LSA. We believe that when stimuli were applied to the afferent division of the facial nerve, they entered the facial motor nuclei; while exiting the nuclei and passing through the REZ, demyelination of the REZ and ephaptic transmission caused by neurovascular conflict spread the electrical stimuli to the entire facial nerve branch. In this model, the vessel itself acts as an electrical bypass and spreads the signal to all branches of the facial nerve; if the vessel is removed from the REZ, no further bypass of electrical stimuli occurs, and the LSR disappears. At OR, electrical currents running through the skin cannot be detected because of electrical resistance of skin. However, if the stimulation intensity exceeds the threshold, the spreading of direct current to other branches of the facial nerve or directly through skin is detected as LSAs, which conduct faster than LSR which runs further pathway. Stimulation intensity and amplitude of the wave are proportional in usual electrophysiological monitoring^18^. While monitoring motor or sensory evoked potentials, as the stimulation intensity increases, more neurons get excited and the amplitude increases proportionally. However, we hypothesized that when the stimulation intensity is increased, the nearby branches of the facial nerve can also be stimulated and that current may spread directly through the skin and form LSAs. This hypothesis can also explain the increase in the LSR amplitude with increasing stimulation intensities and a decrease in the amplitude at higher intensities. As current may spread through the skin or nerves to nearby areas, effective conduction is not possible. Furthermore, direct spreading current may depolarize the affected facial nerve earlier than the current from the LSR pathway; this can be explained as an orthodromic innervation to the brainstem facial nuclei, which then runs through the affected facial nerve branch. Thus, facial nerve branches that are excited early are not excited with LSR because of their refractory period, and thus, show waves of smaller amplitudes.

Increasing the stimulation intensity may become necessary in cases where no definitive LSR is evident. Nevertheless, it is important to acknowledge the potential for it to represent a LSA when the latency period falls below 9.6 ms (ms). Given variations in the parameters and placement of stimulation and recording electrodes among examiners, the absolute threshold of 9.6 ms may not hold universally. Consequently, it is more appropriate not to solely rely on the absolute value of 9.6 ms but rather consider the fact that LSA typically exhibits significantly shorter latency compared to LSR. Consequently, examiners should exercise caution against excessively high stimulation intensities, even in cases where LSR is not distinctly observable.

As the pathophysiology and mechanisms of HFS and LSR remain unclear, there is no definite methodology to measure this electrophysiological sign. Investigating the meaning of these waves will help determine what causes the spasm and why the abnormal muscle response is detected in these patients. In our study group, we applied a new method of LSR detection that involved placing of the anode and cathode in a reverse manner for achieving better outcomes^16^. We have suggested an optimal stimulation intensity range that can diminish the false-positive detection of LSR and make LSR more valuable as a prognostic marker. Further research is required to explore this abnormal electrical response.

### Limitation

Our primary aim was to ascertain an optimal monitoring technique for LSR, emphasizing precise data collection and the reduction of artifact interpretation. It is important to recognize that while our study may not introduce revolutionary alterations to MVD procedures or unveil entirely novel theoretical insights, it serves as a foundational step in the pursuit of recording authentic and dependable LSR. When describing the patterns of AMR in Fig. 6, it's important to note that the amplitudes of each patient varied widely, making statistical analysis unfeasible. Therefore, we could only take a schematic approach to illustrate the patterns.

## Conclusion

In this study, we suggested an optimal stimulation method for the accurate detection of LSR. The optimal stimulation intensity differs for individuals; however, when the intensity surpasses the optimal range for LSR detection, LSAs can be detected with LSR even after successful decompression. The optimal range of stimulation should be obtained by increasing the stimulation intensity in a stepwise manner, but only within the range of onset latency (> 10 ms). When LSR is detected at a onset latency < 10 ms, it is most likely LSA and cannot be trusted.

### Supplementary Information

Supplementary Information 1.

Supplementary Information 2.

## Data Availability

All data generated or analysed during this study are included in this published article and its supplementary information files. Original capture files and video recordings of monitoring LSR is owned by S.K.P. out neurophysiologist. If any request of original EMG data contact the author below. heydaum@daum.net.
