# Supplementary material for: Optimal method for reliable lateral spread response monitoring during microvascular decompression surgery for hemifacial spasm
Source: Sci Rep. 2023 Dec 7;13:21672. doi: 10.1038/s41598-023-49008-1 (PMC10709590; doi:10.1038/s41598-023-49008-1)
Supplement: Supplementary file 2 — Supplementary Information 2. [file 41598_2023_49008_MOESM2_ESM.docx]

Supplementary Materials

[doi.org/10.1159/000330396](https://doi.org/10.1159/000330396)

Samsung Medical Center Grading System for Hemifacial Spasm (SMC Grade)

This grading system, developed by Dr. Park and colleagues, aims to classify patients based on the severity of their hemifacial spasm, which significantly impacts their quality of life. The system consists of four grades, designated as Grade I through Grade IV, each representing a different level of severity. Here is a brief overview of the SMC grading system:

Grade I: Localized spasm primarily around the periocular area.

Grade II: Involuntary movement spreads to other parts of the ipsilateral face, affecting additional muscle groups such as the orbicularis oris, zygomaticus, frontalis, or platysma muscles.

Grade III: The spasm interferes with vision due to frequent tonic spasms.

Grade IV: Disfiguring asymmetry resulting from continuous contraction of the orbicularis oculi muscles, leading to difficulty in eye opening.

This grading system provides a valuable tool for assessing the severity of hemifacial spasm in patients and can aid in determining appropriate treatment approaches.

The different patterns of AMR we have identified through our this study

The first type, pattern A, involved a linear increase of stimuli but a decrease from the peak amplitude. This type of pattern was further sub-divided into the following two patterns. In the first, the AMR amplitude increased on increasing the stimuli but started decreasing or was not detected at higher stimuli. In the second, the AMR amplitude remained similar to the previous pattern observed on increasing the stimuli, but shifted to the right, and AMR was still detected at 30 mA stimulation but with a smaller amplitude. The second type, pattern B, involved a linear but exponential increase at higher stimulation intensities. In the third type, pattern C, the peak amplitude was reached; thereafter, the AMR decreased and disappeared, only to reappear as another wave pattern similar to pattern B at high-intensity stimuli. The fourth type, pattern D, was similar to pattern C but without the disappearance of AMR and consisted of a mix of two separate waves. There were few patients whose response did not match any of these patterns. Schematic representations of the patterns of AMR are provided in Fig. 6.
